# Impacts of electronic device use on adolescents' sexual knowledge, attitude and perception during the COVID-19 pandemic: A representative sexuality survey

**DOI:** 10.3389/fpubh.2023.1034155

**Published:** 2023-03-30

**Authors:** Desiree Man-Sik Tse, Omar Tsui Wai Kiu, Victoria Anna Yeo, Elkie Chan Yu Kiu, Paul Yip, Patrick Ip, Edmond Pui Hang Choi, William Chi Wai Wong

**Affiliations:** ^1^Department of Family Medicine and Primary Care, School of Clinical Medicine, Li Ka Shing Faculty of Medicine, The University of Hong Kong, Hong Kong, Hong Kong SAR, China; ^2^School of Clinical Medicine, LKS Faculty of Medicine, The University of Hong Kong, Hong Kong, Hong Kong SAR, China; ^3^Centre for Suicide Research and Prevention, Hong Kong Jockey Club, The University of Hong Kong, Hong Kong, Hong Kong SAR, China; ^4^Department of Social Work and Social Administration, The University of Hong Kong, Hong Kong, Hong Kong SAR, China; ^5^Department of Paediatrics and Adolescent Medicine, The University of Hong Kong, Hong Kong, Hong Kong SAR, China; ^6^Department of Paediatrics and Adolescent Medicine, Hong Kong Children's Hospital, Kowloon, Hong Kong SAR, China; ^7^School of Nursing, Li Ka Shing Faculty of Medicine, The University of Hong Kong, Hong Kong, Hong Kong SAR, China

**Keywords:** online learning, sex education, sexuality, adolescence, COVID-19

## Abstract

**Background:**

COVID-19 pandemic has led to school closure and social distancing measures for infection control. Many young people thus spent more time on electronic devices and the Internet. This study aimed to determine if and how sexual knowledge, perception and behavior as well as sexuality among Hong Kong adolescents were affected as a result.

**Methods:**

Youth Sexuality Study conducted by The Family Planning Association of Hong Kong (FPAHK) evaluated the sexual knowledge, attitudes and behaviors and sexual health of youth every 5 years since 1981 with adaptations made to the changing environment. We analyzed this cross-sectional data on sexual knowledge, attitude, and experiences as well as the impacts of COVID-19 on daily life, health and relationships. Univariate analysis was conducted to investigate the relationships between the time spent on electronic devices and sexuality, while mediation analyses using the PROCESS procedure were performed to further explore differences in time spent on electronic devices.

**Results:**

During the COVID-19 pandemic, the majority of our participants spent more time on social media and browsing the Internet on electronic devices with less time for extracurricular activities and learning. Nonetheless, there was better overall sexual knowledge and a lower degree of sexual stigma with a higher overall acceptance of sexual minorities. The mediation analyses found that sexual content [Conditional effect = 0.024 (95% CI 0.008, 0.043)] and engagement online [Conditional effect = 0.036 (CI 0.021, 0.053)] could indirectly influence the effect of screen time on the frequency of sexual practices.

**Conclusion:**

Policymakers and frontline professionals should re-examine the pedagogy of the present sex education and consider online sex education as the key mode of delivery while guiding the proper use of electronic devices in the learning and exploration of sexual knowledge.

## Introduction

The COVID-19 pandemic has led to many social distancing measures for infection control such as suspension of non-essential in-person healthcare services and schooling, as well as social gatherings amongst peers and acquaintances. The lack of these face-to-face gatherings and disruption of the school schedules invariably changed their social life and communication, which by extension can affect sexual attitudes and behavior (1–3). In a systematic literature review, it was found that the frequency of sexual intercourse varied depending on co-habitation with fewer casual partners and group sex in Australia and the UK, while the use of sex toys and masturbation increased due to the pandemic (4). Surveys from China, however, reported a reduced frequency of sex, reduced number of partners and increased use of pornography (5). Moreover, age, relationship status and cohabitation with either partner were identified as factors for sexual frequency and satisfaction during the pandemic (6).

While many studies have investigated the changes in sexual behaviors during COVID in the general population, there is little focus on its impacts on adolescents and young people and none explained how the changes could have taken place. The pandemic *per se* could reduce peer contact and in-person schooling, which is important as they are marked by increased demand for social stimulus and peer interaction (7). Subsequently, many of them shifted facets of their daily and social lives to the cyber world (8), exposing them to more sexual content over the Internet. Drawing on the Cultivation Theory, sexual exposures are important to shape the sexual attitudes of consumers (9). It was found that heavier media exposure to sexual content was associated with adversarial sexual beliefs, more permissive sexual attitudes and greater acceptance of uncommitted sexual exploration. Pornography consumption has also been linked to a greater likelihood of perpetrating harassment, aggression, and sexual coercion (10). This was further exacerbated in the Asian context where smartphones and social media usage, alongside the search for sexual content, became more widespread among adolescents (11).

In this study, we aimed to investigate if the pandemic had shaped the sexual knowledge, activities and behavior of adolescents in Hong Kong, thereby aspiring new approaches to improve their sexual well-being and exploring how these effects had influenced the adolescents' relationships and sexual practices. With the pandemic aggravating the use of digital devices, we hypothesized a positive relationship between electronic device usage and sexual attitudes, with increased screen time would be associated with more liberal sexual attitudes (higher acceptance of sexual minorities and various sexual acts). We further hypothesized that those who had spent more time on the Internet would have better sexual knowledge and attitudes, which, in turn, lower stigma and shame toward sexually transmitted infections (STIs).

## Methods

### Procedures

The Youth Sexuality Study (YSS), one of the largest community-based surveys was conducted by the Family Planning Association of Hong Kong (FPAHK) as serial surveillance every 5 years since 1981 to evaluate the sexual knowledge, attitudes and behaviors of the youth and their trends in Hong Kong. The Consumer Search Group (https://www.csg-worldwide.com/about-us/) was commissioned to distribute the questionnaire and collect data. A simple random cluster, stratified by four main districts in Hong Kong (i.e., Hong Kong Island, Kowloon, New Territories West, and New Territories East) was obtained from the Education Bureau to select secondary schools registered in all the districts within the district, and classes from each level of these schools were invited to participate in YSS. Responses were collected from secondary school students from 25 out of 41 selected schools (Response rate of 60.1%). A total of 10,681 responses were received, of which 8,282 responses were from students from Years 7 to 12 (Form 1 to Form 6) in secondary schools across the territory (Response rate of 77.5%). Invitation letters to the parents would be provided at individual schools' request and this project was approved by the FPAHK Ethics Panel (Project No: OA1-2).

### Materials

There were questions assessing participants' demographic and background information including age, gender, education, ethnicity, immigration status and, school and family situations, as well as 15 dichotomous variables designed to measure sexual knowledge (1 = correct and 0 = incorrect/ don't know), adding up to a total of 0 to 15 points. There were 10 items assessing the attitudes toward certain sexual behaviors with 1 = accept and 0 = not accept / don't know. For sexual experience, eight variables with a 4-point Likert scale, ranging from 1 = never to 4 = often, were employed to measure the participants' experience on social media, while 8 other items assessing their personal experience of intimacy and sexual behaviors (0 = never, 1 = 1–5 times and 2 = more than 5 times). Fourteen items were adopted to measure perceived STI-related stigma and shame (12, 13). For the stigma scale, they were asked to rate the nine statements describing people's negative reactions if they had an STI with possible scores from 1 = strongly disagree to 4 = strongly agree, adding up to a total score of 9 to 36. The shame scale had 5 items about different negative emotions that could be present if one had an STI. Participants were told to rate how intense these emotions would be using another 4-point Likert scale ranging from 1 = not at all to 4 = very with the total score ranging from 5 to 20.

In addition, the survey in 2021 collected the participants' responses on the impact of COVID-19. Three and 5 variables were used to assess the youths' health conditions and the quality of their relationships, respectively. Eight questions asking how COVID-19 had affected the participants' daily activities were included. For example, the amount of time spent on electronic devices during the pandemic was assessed by “After the outbreak of COVID-19, how has the amount of time spent on electronic devices been changed?” They were measured using a 5-point Likert scale, ranging from 1 = much reduced to 5 = much increased.

### Data analysis

Descriptive statistics were conducted to characterize the participants' sexual knowledge, attitudes, and experiences, and the impacts of COVID-19 on daily life, health and relationships. The sample was then stratified by the amount of time spent on electronic devices to examine the impact of screen time during the pandemic. Univariate analyses were further conducted to investigate the relationship between participants' sexual health and the change in the amount of screen time during COVID-19, controlled for factors that potentially influenced the outcome variables (i.e., sexual knowledge, attitudes and experiences). A significance threshold of *p* < 0.05 was used and all the analyses were undertaken in SPSS, version 26.

To further delineate the relationships between the impact of time spent on electronic devices, activities over the internet and sexual practices, the mediation analyses using the PROCESS procedure developed by Hayes (14) were carried out. Four items (i.e., caressing sexually, oral sex, vaginal sex and anal sex) were integrated to create a new variable, “sexual practice” as the outcome variable, while the predictor variable for analysis was the time spent on electronic devices during COVID-19 after controlling for age and gender. Full mediation refers to when three conditions are met (15–17) i.e. a significant relationship between the independent variable and the mediator; another significant relation between the mediator and the dependent variable; and an insignificant relation between the independent and dependent variables.

## Results

Table 1 presents the demographics and descriptive data of the participants. More than half (55.4%) of the participants were male. The great majority of them were Chinese (97.9%) born in Hong Kong (80%). Over half (62.8%) of these students were from Junior High (Form 1 to 3). About three-quarters of them (74.8%) were living with their parents whereas another fifth (19.4%) were living with either parent. 61.4% and 55.8% were satisfied with their family and school life, respectively. Under COVID-19, nearly a third (28.2%) felt dissatisfied with their academic performance.

**Table 1:** Demographics, education, and family background of the participants.

	**Mean or count**	**SD**
Age	14.7	1.66
	***n***	**%**
**Gender**		
Male	4,588	55.4
Female	3,694	44.6
**Birthplace**		
Hong Kong	6,628	80.0
China	1,324	16.0
Other places (including Macau and Taiwan)	330	4.0
**Ethnicity**		
Chinese	8,084	97.9
Non-Chinese	175	2.1
**Education**		
Junior high	5,203	62.8
Senior high	3,079	37.2
**Parental situation**		
Currently married	6,520	78.8
Separated or divorced	1,277	15.4
Remarried	339	4.1
Deceased	318	3.8
**Living arrangement**		
Parents	6,185	74.8
Mother alone	1,181	14.3
Father alone	416	5.1
Others	482	5.8
**Satisfaction with family**		
Happy	5,058	61.4
Neutral	2,656	32.3
Unhappy	512	6.2
**Satisfaction with school life**		
Happy	3,622	55.8
Neutral	3,243	39.2
Unhappy	413	5.0
**Satisfaction with academic performance**		
Satisfied	1,450	17.6
Neutral	4,494	54.3
Dissatisfied	2,327	28.2

Table 2 shows the sexual knowledge, attitudes, and behaviors of the participants. The mean score of their sexual knowledge was 8.9 (SD 3.8) out of the total score of 15. The participants' knowledge relating to HPV and pregnancy was particularly poor- scored 1 out of 3 (33.3%); and, 1.8 out of 4 45.0%). The mean stigma score was 24.5 (SD 6.6) and the mean shame score was 14.5 (SD 3.7), both above the 50^th^ percentile (classified as “high”). Acceptance attitudes toward lesbian, gay, bisexual and transgender (LGBT) were moderate at 59.3% toward female homosexuality and 48.8% for transsexuality. Fewer viewed commercial sex positively with 21.7% accepted compensated dating without sex trades; 11.6% accepted compensated dating with sex trades; and, 10.3% accepted being a prostitute. The majority (88.3%) believed masturbation was a normal physiological behavior with 71.3% masturbating in the past week. Our participants had extensive exposure to sexual content on social media: 61.6% were exposed to pornographic content through social media; 61.0% claimed they had gained sex knowledge/ sex education online; and, 59.1% made new friends through social media. Half (51.0%) of them had the experience of caressing sexually but only 8% had sexual intercourse.

**Table 2:** Sexual knowledge, attitudes, and experience of the participants.

	**Mean**	**SD**
Sexual knowledge (overall):	8.9	3.76
HPV Knowledge (3 items)	1	1.11
Pregnancy Knowledge (4 items)	1.8	1.43
STI Knowledge (4 items)	2.5	1.53
AIDS Knowledge (4 items)	2.5	1.56
**Perceived STI-related stigma and shame:**		
Stigma (9 items, score 4–36)	24.5	6.55
Shame (5 items, score 4–20)	14.5	3.71
	Yes (*N*)	%
**Acceptance attitudes of:**		
**LGBT**		
Male homosexuality	4,788	57.9
Female homosexuality	4,943	59.8
Bisexuality	4,810	58.2
Transsexuality	4,030	48.8
**Commercial sex**		
Compensated dating without sex trades	1,797	21.7
Compensated dating with sexual transactions	958	11.6
Being a prostitute	851	10.3
**Masturbation**		
Normal physiological phenomenon	5,718	88.3
Harmful to physical health	2,378	40.4
Harmful to mental health	2,350	39.9
Past week's experience of masturbation:	2,014	71.3
**Experience of:**		
Holding hands	1,775	79.3
Hugging	1,634	73.1
Caressing non-sexually	808	36.1
Caressing sexually	1,138	51.0
Kissing	376	16.8
Oral sex	236	10.6
Sexual intercourse	179	8.0
Anal sex	64	2.9
**Experience on social media:**		
Exposure to pornographic content	5,083	61.6
Search of sex information	5,033	61.0
Making new friends	4,881	59.1
Dating people	1,718	20.8
Discussing sex	3,684	44.8
Naked chat	397	4.8
Sexual relationship	573	6.9
Image-based sexual violence	537	6.5

**Figure 1:**
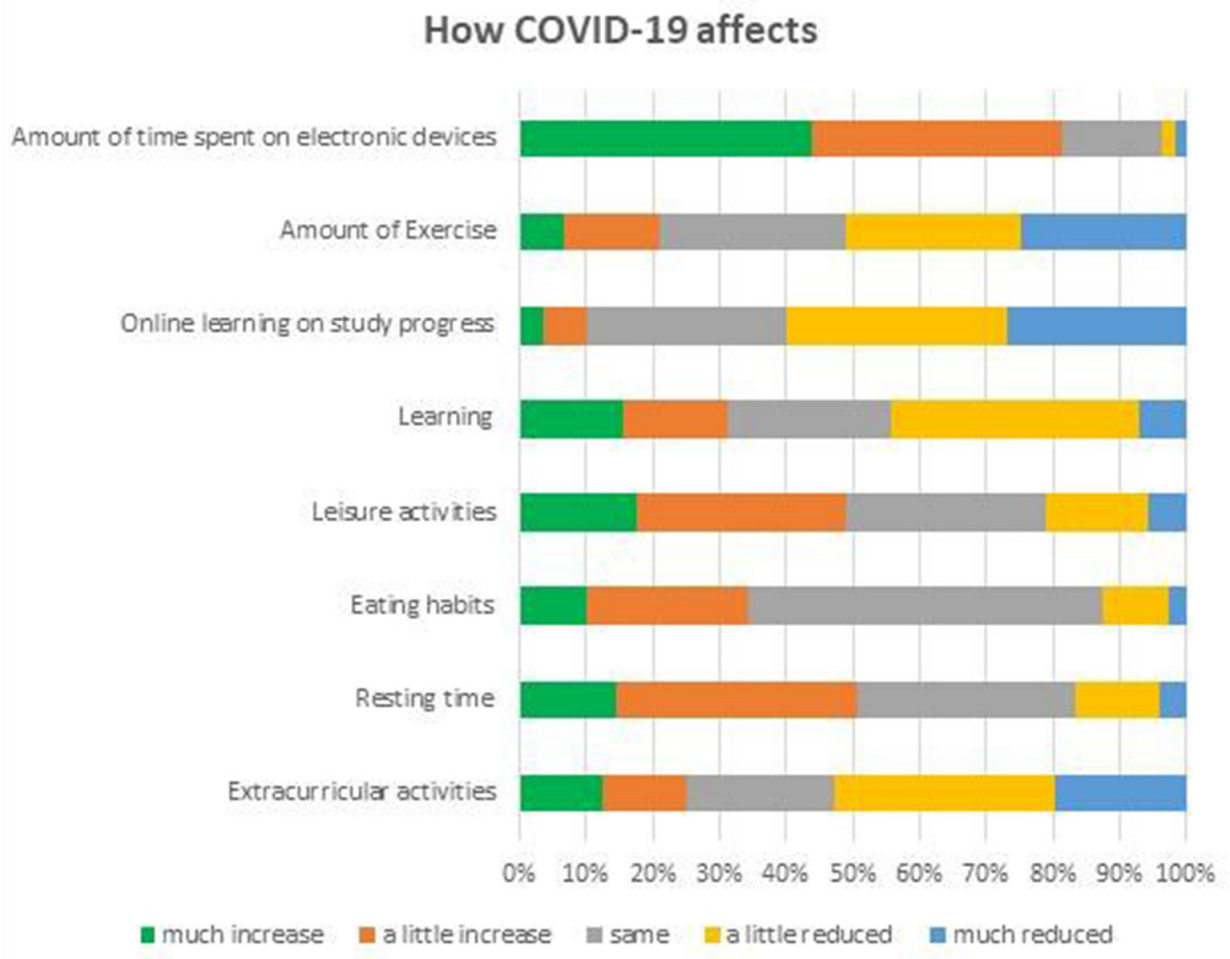
The effect of COVID-19 on the participants' daily life and health.

**Figure 2:**
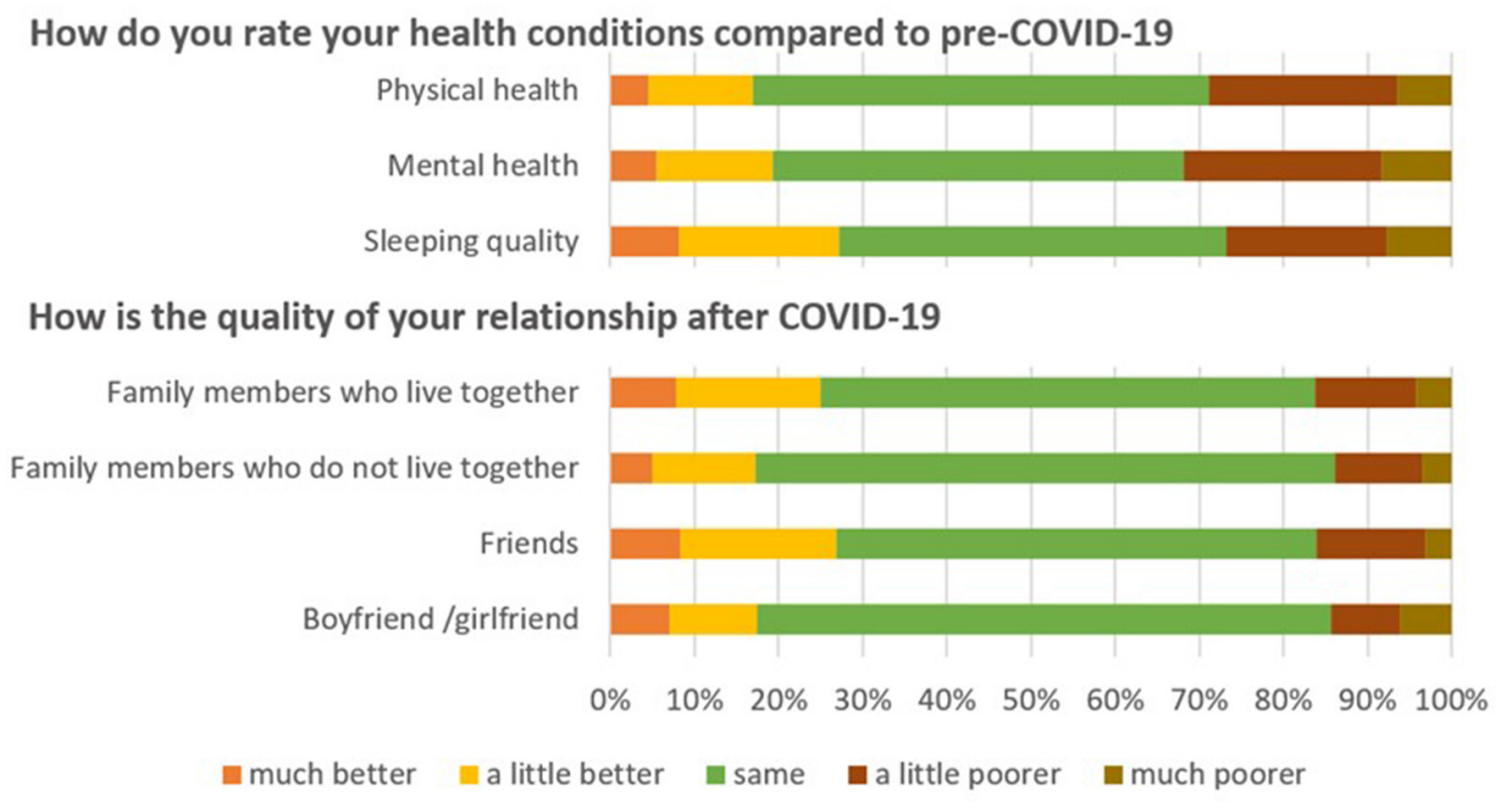
The effect of COVID-19 on the participants' relationship.

Figures 1, 2 describe the effect of COVID-19 on participants' daily life, health and relationships. They reported that the pandemic had reduced their study progress with online learning (49.8%); the amount of exercise (30.0%); time spent on extracurricular activities (27.6%); and, overall learning experience (13.1%). Simultaneously, they reported an increase in time on electronic devices (77.4%); resting (34.0%); and, leisure activities (28.0%). Most reported that their health conditions and relationships with their “significant others” i.e., family members, friends, boyfriend, or girlfriend largely remained unchanged when compared to pre-COVID-19.

The relationships between the impact of time spent on electronic devices during COVID-19 and sexual knowledge, attitudes and behaviors were depicted in Table 3. Participants who spent an increased amount of screen time scored significantly higher on sexual knowledge, in general, and on specific sex-related topics. Their acceptance attitudes toward LGBT were also consistently and significantly higher than those spent less screen time during the pandemic. More sexual experiences for social media including more exposure to pornography; more search of sex information; more social acquaintances online; and more sexual discussion, were reported. In contrast, those spent more time online had significantly lower STI stigma and shame.

**Table 3:** Univariate analysis of time spent on electronic device on sexuality/sexual health during COVID-19 after controlling for gender, age, satisfaction with school life and academic performance.

	**The amount of time spent on electronic devices during COVID-19**		
	**Reduced/same**	**Increased**	
	**Mean**		**p**
**Sexual knowledge (overall)**	8.0	9.2	<.001
HPV Knowledge (3 items)	0.8	1.0	<.001
Pregnancy Knowledge (4 items)	1.5	2.0	<.001
STI Knowledge (4 items)	2.0	2.6	<.001
AIDS Knowledge (4 items)	2.1	2.7	<.001
**Perceived STI-related**			
Stigma (9 items, score 9–36)	24.7	24.1	<.001
Shame (5 items, score 5–20)	14.7	13.9	<.001
	%	%	p
**Acceptance attitudes of:**			
**LGBT**			
Male homosexuality	47.2	61.8	0.002
Female homosexuality	49.6	63.6	<.001
Bisexuality	47.0	62.2	<.001
Transsexuality	40.5	51.7	<.001
**Commercial sex**			
Compensated dating without sex trade	20.5	22.3	<.001
Compensated dating with sex trade	12.5	11.4	0.073
Being a prostitute	10.3	10.3	0.024
**Masturbation**			
normal physiological phenomenon	82.0	89.9	0.138
harmful to physical health	43.5	39.6	<.001
harmful to mental health	43.0	39.0	<.001
**Past week experience of masturbation:**	70.8	71.3	0.001
**Experience of:**			
Holding hands	77.5	79.4	0.284
Hugging	72.9	74.5	0.313
Caressing non-sexually	36.1	36.6	0.064
Caressing sexually	16.5	18.3	0.253
Kissing	50.4	54.2	0.056
Oral sex	10.2	11.6	0.301
Sexual intercourse	7.6	9.9	0.790
Anal sex	3.0	1.7	0.217
**Experience on social media:**			
Exposure to pornographic content	50.2	65.4	<.001
Search of sex information	49.9	64.6	<.001
Making new friends	52.2	61.5	<.001
Dating people	24.2	20.1	<.001
Discussing sex	40.7	46.1	<.001
Naked chat	9.9	3.5	<.001
Sexual relationship	12.7	5.5	<.001
Image-based sexual violence	11.3	5.3	<.001

**Figure 3:**
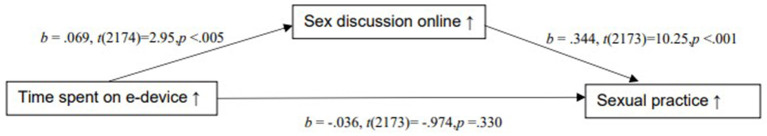
Mediation analysis investigating the effect of time spent on electronic devices on sexual practices, mediated through sexual discussion online.

**Figure 4:**
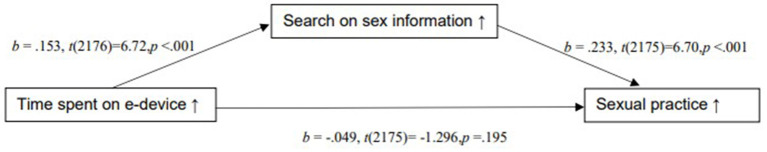
Mediation analysis investigating the effect of time spent on electronic devices on sexual practices, mediated through sexual knowledge.

The results of the mediation analyses were depicted in Figures 3, 4. Figure 3 shows that discussing sex with others on social media would mediate the relation between the time spent on electronic devices and sexual practices. The negative relation of the impact of screen time online on sexual practice would become positive when the mediator sex discussion was involved [conditional effect = 0.024 (95% CI 0.008, 0.043)]. For those who had spent more time on their electronic devices, they were also involved more in sexual discussions on social media, hence, more likely to have sexual practice. Moreover, screen time online had an indirect effect on youths' sexual practice through the mediator of sex discussion on the internet. Figure 4 demonstrates the same pattern with these relationships with another mediator, which was the exposure to sexual knowledge on the Internet with conditional effect = 0.036 (CI 0.021, 0.053).

## Discussion

### Electronic device use and adolescent sexuality

In the current study, we found that participants who had reported increased usage of electronic devices had consistently higher mean scores in sexual knowledge as well as specific topics of reproductive health. While COVID-19 diverted time away from school to electronic devices, interested students were free to explore sexual topics on the Internet, improving their sexual knowledge. While it is unclear how unstructured exploration of online sexual information would affect young people's views on these issues, the effectiveness of targeted online sex education is well documented (18, 19). Sexual health technologies such as text messages or mobile phones and applications have shown positive effects on sexual education amongst adolescents in the United States (20). About 84% of the young people in South Africa, Nigeria and Kenya also considered that social media was a reliable source of sexual health communication (21).

Concomitant with the increase in sexual knowledge was a decrease in the perceived stigma and shame scores, as well as more accepting attitudes toward LGBT within the same group. Cunningham and colleagues found that STI-related stigma was negatively associated with the disclosure of sexual history to healthcare providers, creating a barrier to seeking medical assistance and a hindrance to the doctor-patient relationship among young people (22). In conjunction with our results, this suggested that electronic device usage help lower the stigma associated with STIs, which could affect youth's likelihood of seeking STI-related care, thus offering the potential for early healthcare intervention.

In line with the findings before the pandemic, youths in Hong Kong held a more liberal sexual attitude (23). The acceptance of various sexual behaviors and LGBT communities were high, particularly so among the participants who spent more time on electronic devices. Compared to televised sexual materials, the multi-media sexual content available on the Internet is simply too vast to be quantified. Research on the level of sexual content on television could provide some insights to explain the impact of time spent on electric devices on adolescents' sexual attitudes. Based on content analyses, the prevalence of sexual content in mainstream media ranged from 82 to 85% (24, 25). Such media exposure had moderate effects on sexual attitudes in youths, associated with increased liberal sexual attitudes that led to more risky sexual behaviors, such as multiple sex partners and earlier sexual initiation (26). Brown and Newcomer further discovered a bidirectional relationship between television viewing patterns and the sexual status of youths (between virgin and sexually active ones), in which sexually active adolescents had viewed more sexual content and the heavier viewers were more likely to have had sexual intercourse. This leads to some scholarly discussions of whether sexual content was the cause or result of sexual activity (27).

To further explain the effect of media on sexual practice, we explored the attention to sexual content online and found that it had a mediating effect on adolescents' sexual practice. Our findings suggest that time spent on electronic devices *per se* does not affect sexual practice. Rather, the effects of screen time depend on the sexual content that the viewers choose to attend to. Our results also implied when teaching was moved to virtual platforms during school closure, some students would spend more time on the Internet learning while others might spend an equivalent amount of time on sexual materials. As it is ineffective (and not feasible) to control access to sexual materials by policing the media on a mass scale, policymakers should be encouraged to take advantage of digital technologies to provide comprehensive sex education online. The United Nations Sexual and Reproductive Health Agency (UNFPA) has been working with governments to implement comprehensive sex education, making good use of social media and smartphones to promote digital sexual education, delivering accurate knowledge, values and attitudes during school closures (28). Education on safe sex practice and age at sex debut is particularly crucial for shaping adolescents' perception of sex and sexual health.

### Significance of the study

The study is the first of its kind focusing on the impact of sexual knowledge, attitudes and behaviors of secondary school students during the COVID-19 pandemic, emphasizing the effects of media usage during the suspension of face-to-face learning in Chinese society. The secondary school students reported that their learning is drastically affected by the pandemic, yet we found the sexual knowledge and tolerance toward LGBTQ of students might have improved by the sexual content available on the Internet featuring same-sex marriages and the associated legal battles in certain countries, as well as the activities promoted by the LGBT communities. In contrast, only 59.3% of secondary school students scored all items about HPV, pregnancy, STIs and HIV correctly, down from 67% in the same survey conducted in 2016 (29). Such mixed findings suggest that e-learning could be leveraged to promote sexual inclusivity as an alternative to traditional classroom teaching.

Generally, sexuality is not openly discussed in a Chinese society such as Hong Kong. Sex education is not a core subject in the formal curriculum (30). The syllabus varies in different schools depending on its principles, missions, ideology and resources. As it is not an examinable subject, students' sexual knowledge is not formally assessed (30). However, the declining trends in sexual knowledge demonstrated the deficiency of sexual education in Hong Kong. Other than traditional school-based sexuality education, our findings suggest that sex education could be conducted online constructively, of which its effectiveness should be monitored and evaluated in a timely manner. For example, sex education conducted through the Internet in Shanghai could increase the median scores for overall knowledge, as well as that of specific aspects of reproductive health such as STIs (31). Corroborating the findings of Ward and others, the relationship between electronic device usage and the frequency of sexual practice indicated that sexual materials provided online could increase the risk of sexual permissiveness and sexual violence victimization (9, 32). Therefore, policymakers should consider how to overcome the spread of sexual misinformation before promoting online learning as an alternative to traditional sex education.

The impacts of COVID-19 on adolescents' attitudes, knowledge and behaviors toward sex should not be underestimated. While sex education is delivered in-person by trusted educators in school traditionally, changes could take place when learning modalities are switched from face-to-face to online learning. Given the strong correlation between the usage of electronic devices and improved sexual knowledge as well as the increased tolerance of alternative sexual expression, at least more online elements should be integrated into the classroom teachings of sex education. It is essential to consider that most online sexual information is unregulated, leading them vulnerable to false sexual knowledge and practice. Tarzia and Tyler have identified links between intimate partner sexual violence (IPSV) and the mainstreaming of pornography (33). They also found associations between pornography consumption and risky sexual behaviors such as infrequent condom use in Sweden (34). All the above call for concerted efforts to provide a safe online environment for adolescents to explore sex and sexual health.

### Limitations

Despite efforts in random cluster sampling, anonymization and the diversification of question responses, a few limitations may impede the interpretation of our data. Our study employed a self-reported questionnaire therefore could be prone to self-report bias. Furthermore, there might be residual confounding factors such as technological advancement and changes in social norms, which means sampling bias toward more economically privileged adolescents with access to electronic devices. Nonetheless, we recruited participants across the territory so our samples were representative of the youth population in Hong Kong. For the impact of the pandemic, we could only determine its influence on sexuality based on the participants' subjective perceptions due to the lack of data before the pandemic, although the questionnaire was updated every 5 years to reflect changes in society and is germane to contemporary social conditions. Other weaknesses may include unmeasured confounders that potentially affect the veracity of the statistical analyses. Future studies should consider the links between various online experiences such as the types of pornography and the sexual satisfaction of young adults. Interventional studies may help to clarify the causal relationship between time spent on electronic devices and changes in sexuality.

## Conclusion

Under the COVID-19 pandemic, an increase in screen time is found to be related to improved sexual knowledge, reduction in sexual stigma and higher acceptance of LGBTQ. However, sexual content over the Internet can also lead to more sexual practices. Schools should re-evaluate the pedagogy of the present sex education and introduce online learning elements to provide suitable guidance on the proper use of electronic devices in the learning and exploration of sexual knowledge. We advocate that online comprehensive sex education is feasible for improving sexual knowledge and increasing tolerance toward alternative sexual expressions, thereby empowering adolescents to make informed decisions on sexual behaviors. Social media offers tremendous potential for sexual education as a complementary tool to classroom teachings in Hong Kong. Educators, sexual scientists and policymakers should make a concerted effort to implement a feasible curriculum for sexual education in the post-pandemic era.

## Data availability statement

The raw data supporting the conclusions of this article will be made available by the authors, without undue reservation.

## Ethics statement

The studies involving human participants were reviewed and approved by FPAHK Ethics Panel. Written informed consent from the participants' legal guardian/next of kin was not required to participate in this study in accordance with the national legislation and the institutional requirements.

## Author contributions

WW led the project and DT was primarily responsible for the results and manuscript preparation. OK, VY, and EK assisted in preparing the manuscript. PI and PY were key collaborators who edited the manuscript. PI, PY, and EC provided guidance and advice on the preparation of the manuscript. All authors approved the final manuscript and accepted the responsibility for submitting it for publication.
